# The natural compound neobractatin inhibits tumor metastasis by upregulating the RNA-binding-protein MBNL2

**DOI:** 10.1038/s41419-019-1789-5

**Published:** 2019-07-18

**Authors:** Juan Zhang, Zhaoqing Zheng, Man Wu, Li Zhang, Jing Wang, Wenwei Fu, Naihan Xu, Zhili Zhao, Yuanzhi Lao, Hongxi Xu

**Affiliations:** 1 School of Pharmacy, Shanghai University of Traditional Chinese Medicine, 201203 Shanghai, P.R. China; 2 Engineering Research Center of Shanghai Colleges for TCM New Drug Discovery, 201203 Shanghai, P.R. China; 3 Key Lab in Healthy Science and Technology, Division of Life Science, Graduate School at Shenzhen, Tsinghua University, 518055 Shenzhen, P.R. China

**Keywords:** Breast cancer, Collective cell migration

## Abstract

Tumor metastasis is the predominant cause of lethality in cancer. We found that Neobractatin (NBT), a natural compound isolated from *Garcinia bracteata*, could efficiently inhibit breast and lung cancer cells metastasis. However, the mechanisms of NBT inhibiting cancer metastasis remain unclear. Based on the RNA-sequencing result and transcriptome analysis, Muscleblind-like 2 (MBNL2) was found to be significantly upregulated in the cells treated with NBT. The Cancer Genome Atlas (TCGA) database analysis indicated that the expression of MBNL2 in breast and lung carcinoma tumor tissues was significantly lower compared to normal tissues. We thus conducted to investigate the antimetastatic role of MBNL2. MBNL2 overexpression mimicked the effect of NBT on breast cancer and lung cancer cell motility and metastasis, in addition significantly enhanced the inhibition effect of NBT. MBNL2 knockdown furthermore partially eliminated the inhibitory effect of NBT on metastasis. Mechanistically, we demonstrated that NBT- and MBNL2-mediated antimetastasis regulation significantly correlated with the pAKT/epithelial-mesenchymal transition (EMT) pathway. Subsequent in vivo study showed the same metastasis inhibition effect in NBT and MBNL2 in MDA-MB-231 xenografts mouse model. This study suggest that NBT possesses significant antitumor activity in breast and lung cancer cells that is partly mediated through the MBNL2 expression and enhancement in metastasis via the pAKT/EMT signaling pathway.

## Introduction

Breast cancer (BC) and lung cancer (LC) are common lethal malignancies with poor prognoses^1,2^, primarily due to metastasis. Cancer metastasis involves local invasion by the primary tumor, followed by circulatory system entry and distant organ infiltration^3–5^. The initial step in metastasis is the attainment of invasive capability by the tumor cells.

Natural compounds have long been recognized as valuable sources for drug development^6–8^. *Garcinia species* have been studied for more than 70 years, and many bioactive compounds with anticancer potential have been identified^9^. Our previous study demonstrated that the antimetastatic effect of Guttiferone K (GUTK), isolated from *Garcinia yunnanensis*, resulted from restoration of profilin 1 levels, which are aberrantly reduced in cancer cells^10^. Oblongifolin C (OC), a polycyclic polyprenylated acylphloroglucinol (PPAP) purified from G. *yunnanensis* Hu, inhibits metastasis in esophageal cancer and hepatocellular carcinoma by upregulating keratin 18 and tubulins^11^; OC is also an autophagic flux inhibitor that blocks autophagosome-lysosome fusion and autophagic degradation^12^.

In recent studies, we have shown that Neobractatin (NBT), a natural compound isolated from *Garcinia bracteata* by C. Y. Wu ex Y. H. Li, possesses anti-tumor activities, such as inducing cell apoptosis and inhibiting autophagic flux^13^. In this study, we also found that NBT exhibited potent anti-metastatic activity against BC and LC both in vitro and in vivo. High-throughput RNA-sequencing (RNA-seq) results showed that NBT upregulated the RNA-binding protein MBNL2. Muscleblind-like (MBNL) proteins constitute a family of RNA-binding factors that regulate developmentally programmed alternative splicing in multiple organs and have been studied in brain, heart, and muscle tissues^14–16^. Accumulating evidence suggests that MBNL proteins play critical roles in tumorigenesis^17^. It has been shown that MBNL1 suppresses breast cancer metastatic colonization through regulating transcript stabilization^18^. A recent study showed that MBNL1 inhibited colorectal cancer metastasis by modulation of the Snail/E-cadherin axis^19^. Furthermore, another family member MBNL3, was found to act as an oncogene, because it promoted tumorigenesis and its high expression indicated poor prognosis in hepatocellular carcinoma^20^. Analysis of the TCGA database showed that Muscleblind-like protein 2 (MBNL2) was expressed to a greater extent in normal tissues than in breast and lung tumors. This finding led us to hypothesize that targeting MBNL2 may impair cancer metastasis.

In this study, we found that NBT significantly inhibited cancer metastasis both in vitro and in vivo and identified MBNL2 as a mediator of NBT’s effect on cell metastasis. These findings suggest that targeting MBNL2 with NBT may serve as a potential anti-metastatic therapeutic strategy in human breast and lung cancer.

## Results

### NBT inhibits the metastasis in MDA-MB-231 and A549 cells

**Fig. 1:**
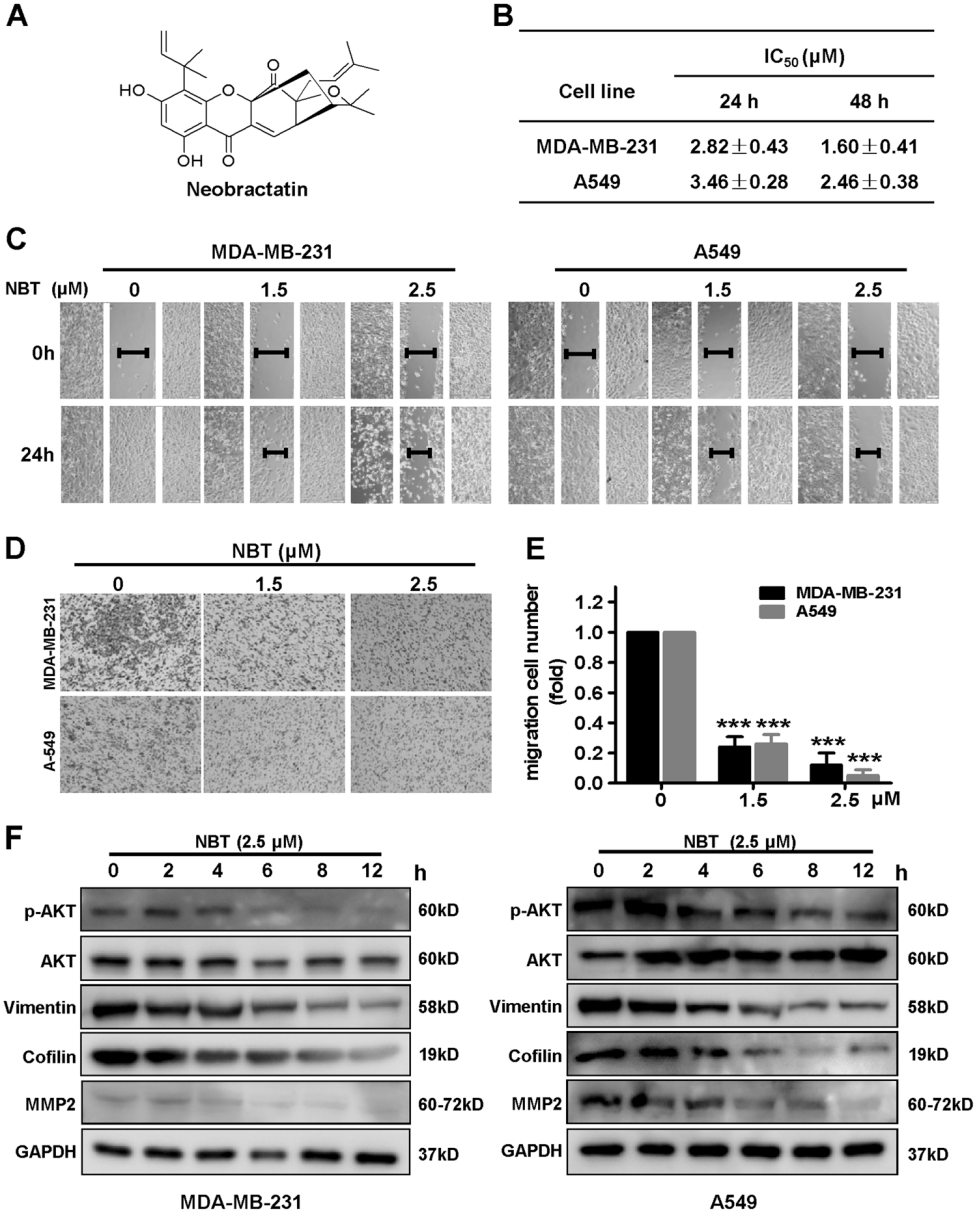
Neobractatin (NBT) inhibits metastasis in MDA-MB-231 and A549 cells. **a** Chemical structure of NBT. **b** Cell viability assays. The MTT assay was used to examine the cell viability in the absence or presence of different doses of NBT for 24 h. **c** Wound healing assay. A scratch was made in monolayers of MDA-MB-231 and A549 cells, and the migration ability of the cells treated with or without different concentrations of NBT was monitored with an inverted microscope. **d** Cell migration measured by transwell assays. The cells were treated with different concentrations of NBT for 24 h, and migrated or invaded cells were fixed and stained with 0.1% crystal violet. **e** The summary of data for transwell migration assays. **f** Western blot asssay. MDA-MB-231 and A549 cells were treated with NBT (2.5 μM) for indicated time points. Data shown are mean ± SD of three independent experiments. ***P < 0.001

Our previous studies have shown that the natural compound NBT (chemical structure shown in Fig. 1a), isolated from *Garcinia bracteata*, exhibited potent antitumor activities^13^; however, the mechanism of its anti-tumor action is unclear. Tumor metastasis is the predominant cause of lethality in cancer. We therefore investigated the effects of NBT on tumor metastasis in highly metastatic human BC and LC cells. As shown in Fig. 1b, NBT decreased the viability of MDA-MB-231 and A549 cells, with IC_50_ values of 2.82 ± 0.43 and 3.46 ± 0.28 μM, respectively. NBT also suppressed the migration of MDA-MB-231 and A549 cells in a concentration-dependent manner in the wound healing assay (Fig. 1c). To confirm the effect of NBT on cell migration, we examined whether NBT inhibited migration in the transwell assays. As shown in Fig. 1d, e, NBT reduced the number of migrated cells in a dose-dependent manner. These results indicated that treatment with NBT markedly inhibited metastasis of MDA-MB-231 and A549 cells in vitro without apparent cytotoxicity. To explore the potential mechanism of action for NBT, We then measured the protein levels associated with the metastasis-related protein. As shown in Fig. 1f, NBT decreased the expressions of pAKT, the epithelial-mesenchymal transition (EMT) marker vimentin, cofilin, and MMP2 in a time-dependent manner in MDA-MB-231 and A549 cancer cells, the statistic analysis was shown in Fig. S1a. Similarly, protein levels of the pAKT, vimentin, cofilin, and MMP2 were decreased in a dose-dependent manner (Fig. S1b). Together, these results suggested that NBT suppressed cancer cell metastasis in vitro, possibly through targeting the pAKT/EMT signaling pathways.

### NBT suppresses the pulmonary metastasis of BC cells in vivo

**Fig. 2:**
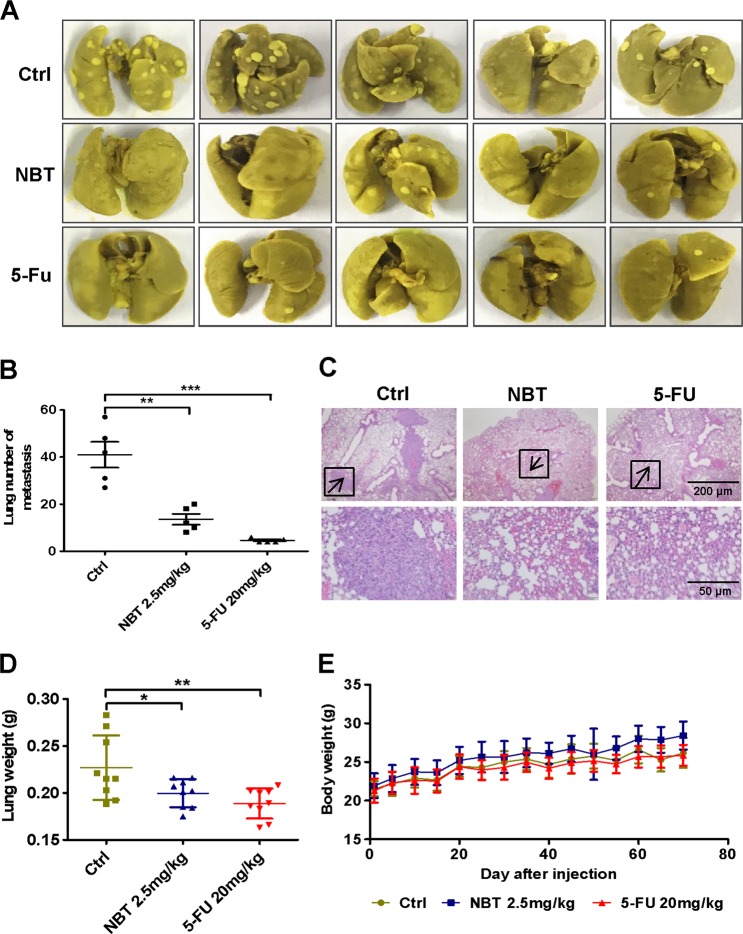
NBT exhibits potent antitumor activity in a metastasis mouse model. Six-week-old female nude mice were injected with 1 × 10^6^ MDA-MB-231 cells in the tail vein. After injection, the mice were divided into three groups (n = 8) and administered DMSO (vehicle group), NBT (2.5 mg/kg per days), or 5-FU (20 mg/kg per 2 days) using intraperitoneal injection. **a** Representative lung images taken from mice at 2 months. **b** Quantitative analysis of metastatic nodes in the lung. **c** Representative HE-staining of lung tissues from the three groups. **d** Lung weight analysis after treatment. **e** Body weight analysis every other day throughout the entire experiments. *P < 0.05, **P < 0.01, ***P < 0.001

We next examined the in vivo effect of NBT using a pulmonary metastasis mouse model. As shown in Fig. 2a, b, lung tumor nodules were present in all groups, but both NBT and 5-FU reduced the number and size of the nodules, as also observed in HE-stained tissue Fig. 2c. Additionally, the weight of the lungs in the groups treated with NBT and 5-FU was significantly smaller than in the control group (Fig. 2d). NBT did not cause significant side effects in mice and only slightly reduced the body weight (Fig. 2e). Other tissues, such as heart, liver, spleen, and kidney, did not show obvious morphological changes (Fig. S5a). In summary, our in vivo study indicated that NBT suppressed pulmonary metastasis of BC cells without significant side effects in nude mice.

### MBNL2 is the potential target of NBT

**Fig. 3:**
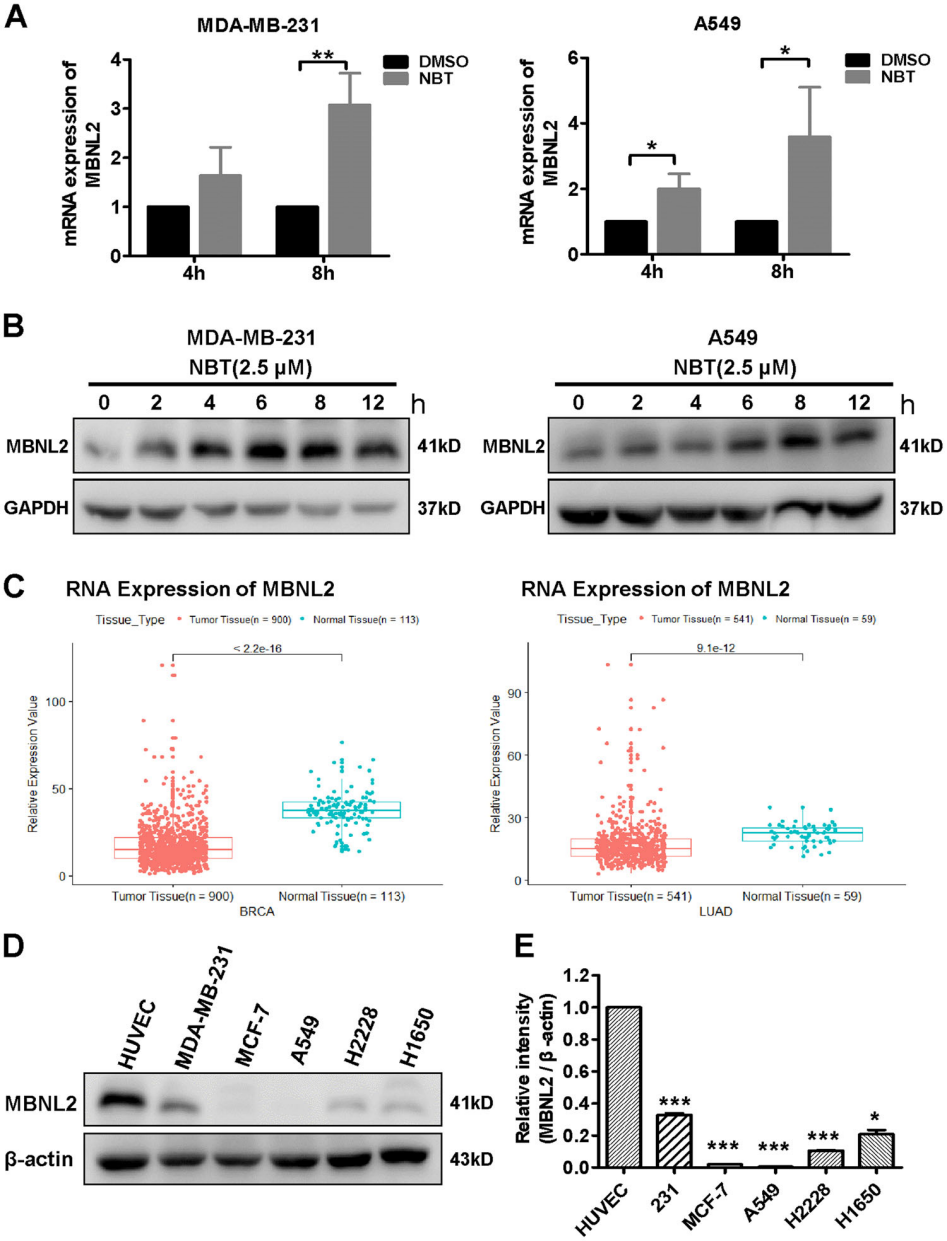
MBNL2 is upregulated in breast cancer and lung cancer. **a** MDA-MB-231 and A549 cells treated with NBT (2.5 μM) for 4 h and 8 h analyzed by real-time RT-PCR. **b** MDA-MB-231 and A549 cells treated with 2.5 μM NBT for different times analyzed by western blotting. **c** Scatter diagram derived from gene expression data in TCGA comparing the expression of MBNL2 in tumor tissue and normal tissue in breast and lung cancers. **d** MBNL2 expression detected by western blot analysis in Human Umbilical Vein Endothelial Cells (HUVEC), breast cancer MDA-MB-231 and MCF-7 cell lines, and lung cancer A549, H2228, and H1650 cells, with β-actin used as a loading control. **e** Statistical analysis from western blotting. The data are expressed as the mean ± SD of at least three independent experiments.*P < 0.05, **P < 0.01, ***P < 0.001

To investigate in more detail the mechanism by which NBT inhibits metastasis, we performed an RNA-sequencing study to search for potential targets of NBT (data not shown). We found that NBT treatment increased the mRNA level of MBNL2 remarkably. MBNL2 belongs to the Muscleblind-like family and contributes to myotonic dystrophies type 1 and 2 (DM1 ad DM2) and expresses in the hippocampus and MBNL2 knockouts show a decrease in *N*-methyl-D-aspartic acid receptor (NMDAR) synaptic transmission and impaired hippocampal synaptic plasticity. Major pathological features of the DM brain result from disruption of the MBNL2-mediated developmental splicing program^15,21^. To confirm the results of RNA-sequencing, we performed real-time PCR and western blot assays to examine the mRNA and protein levels of MBNL2 in NBT-treated MDA-MB-231 and A549 cells. We found that both the protein and the mRNA levels of MBNL2 were elevated in MDA-MB-231 and A549 cells after NBT treatment for 8 h (Fig. 3a, b and Fig. S2a). We also found that the level of MBNL2 protein increased in the lung tissues of the NBT and 5-FU treated groups (Fig. S2b). Furthermore, we examined the effect of NBT on the distribution of MBNL2 in A549 cell line. Immunofluorescence assay showed that NBT treatment increased the fluorescent intensity compared with vehicle treatment (Fig. S2c).

Recent studies have reported that MBNL2 had a role in EMT, which is important in tumor metastasis^17^, suggesting that MBNL2 might play a pivotal role in the invasive properties of cancer cells. To further elucidate the relationship between MBNL2 and BC or LC, we performed a clinical study with the original data in TCGA. We found that MBNL2 was lower expressed in both breast carcinoma and lung adenocarcinoma compared to the non-cancerous tissues (Fig. 3c). We also examined the expression of the MBNL2 protein in human BC cells, LC cells, and human umbilical vein endothelial cells (HUVEC, normal cells). Western blot indicated that the level of the MBNL2 protein in cancer cells was much lower than that in HUVEC normal cells (Fig. 3d, e).

### A role for MBNL2 in the pathogenesis of BC and LC cells

**Fig. 4:**
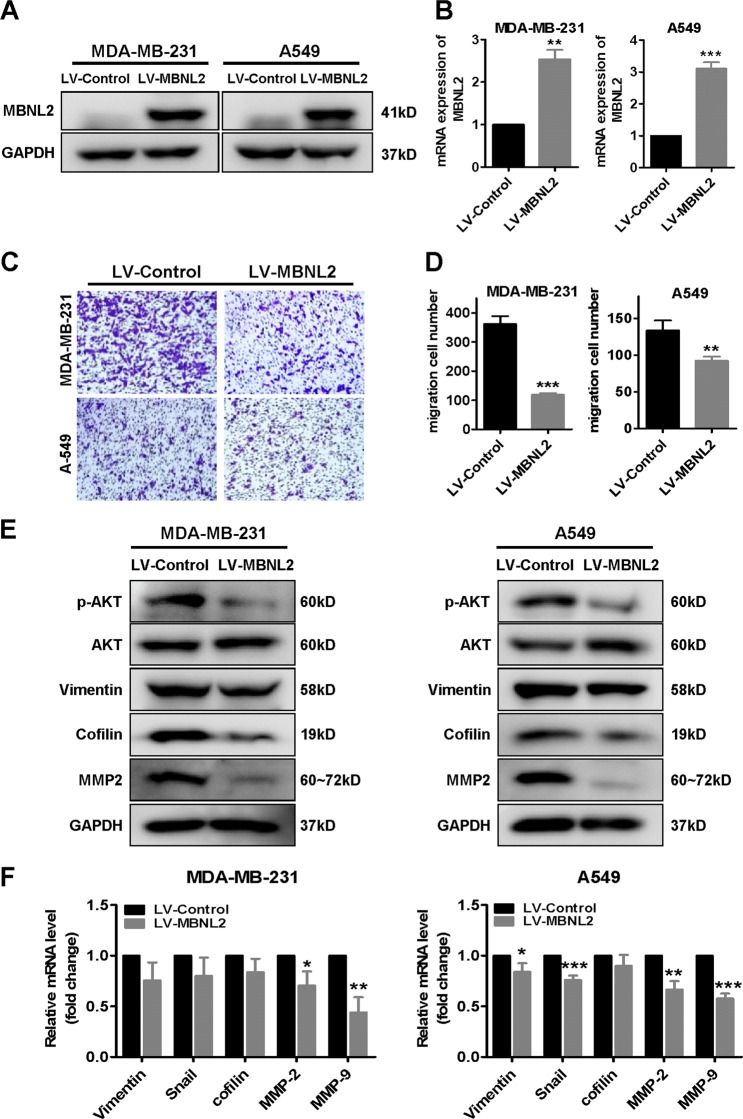
Overexpression of MBNL2 suppresses cancer metastasis. **a**, **b** The levels of MBNL2 protein (**a**) and mRNA (**b**) in MDA-MB-231 and A549 cells stably overexpressing MBNL2. LV-MBNL2, recombinant lentivirus overexpressing MBNL2; LV-control, recombinant lentivirus negative control. **c** Detection of cell migration using a transwell assay in MDA-MB-231 and A549 cells transduced with lentiviruses expressing either MBNL2 plasmid or empty vehicle. The cells were stained with crystal violet and imanged by microscope. **d** Counts of cell number from transwell assay in each group. **e** Western blot analysis of metastasis-related proteins in MDA-MB-231 and A549 cells transduced with lentiviruses expressing either MBNL2 plasmid or empty vehicle. **f** qRT-PCR analysis of the mRNA expression levels for proteins relevant to EMT in MBNL2 overexpressing MDA-MB-231 and A549 cells. The data are shown as the mean ± SD of three replicates. *P < 0.05, **P < 0.01, ***P < 0.001

Since the expression of MBNL2 is low in cancers, we examined the relationship of the level of MBNL2 to metastasis in BC and LC. We induced MBNL2 overexpression in MDA-MB-231 and A549 cells using transduction with an MBLN2-lentivirus construct. The protein and mRNA levels of MBNL2 were successfully upregulated in both MDA-MB-231 and A549 cells (Fig. 4a, b). The transwell migration assays indicated that overexpression of MBNL2 significantly decreased cell migration in MDA-MB-231 and A549 cells compared with control cells transfected with empty vector (Fig. 4c, d). We also investigated the effect of MBNL2 overexpression on cell proliferation. The colony-formation assay indicated that overexpression of MBNL2 did not alter the growth of either BC or LC cells (Fig. S3a, b). The expression levels of some key tumor metastasis-related proteins including phospho-AKT, vimentin, cofilin, and MMP-2 decreased in MBNL2 overexpressed cells (Fig. 4e and Fig. S3c). The mRNA levels of these genes were also significantly reduced in MDA-MB-231 and A549 cells that overexpressed MBNL2 (Fig. 4f). These results demonstrated that overexpression of MBNL2 inhibits tumor metastasis, which might be associated with effects on the pAKT/EMT signaling pathway.

### High expression levels of MBNL2 in BC cells prevents metastasis in vivo

**Fig. 5:**
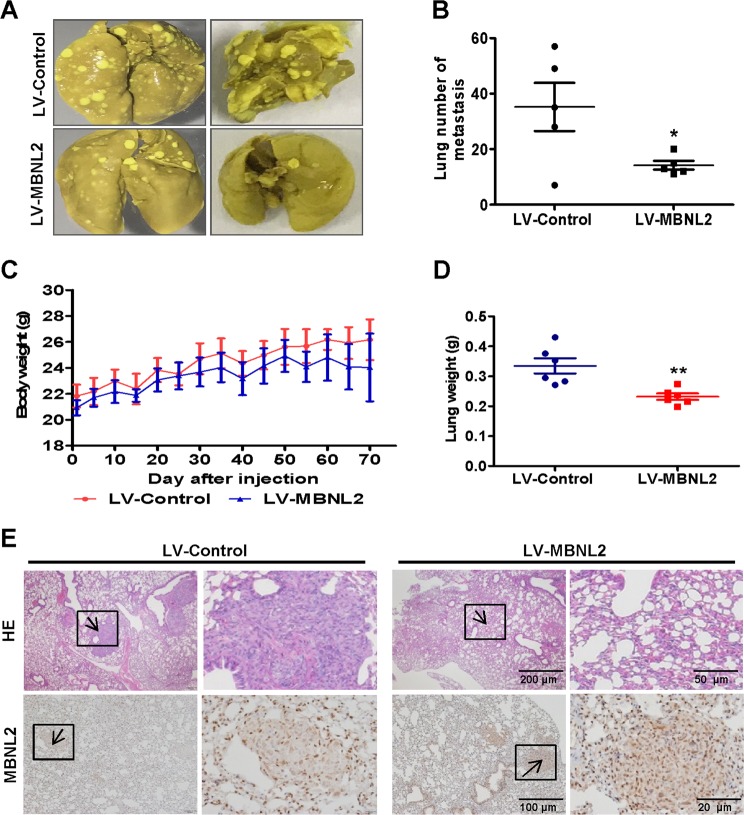
MBNL2 overexpression attenuates the metastatic ability of breast cancer in vivo. **a** Representative examples of lungs from each group (*n* = 6) after 2 months. **b** Quantitative analysis of metastatic nodes in the lung. **c**, **d** Body weight and lung weight analysis after injected throughout the entire experiments. **e** HE & immunohistochemistry staining of MBNL2 in lung tissues. Data are presented as mean ± S.D. *P < 0.05, **P < 0.01

To examine the in vivo effect of MBNL2 on tumor metastasis, we designed a metastasis model by tail vein injection of MBNL2-overexpressing BC cells. The results showed that the number of metastatic nodules on the surface of the mouse lungs was markedly decreased in BC cells that overexpressed MBNL2 (Fig. 5a, b). We also found that the weight of lungs was lower in animals after injection of MBNL2-transduced BC cells than in controls (Fig. 5d), while there were no significant differences in body weight between the LV-control and MBNL2-transduced animals (Fig. 5c). To confirm that the nodules were metastatic tumors, staining with HE was used to examine the lung tissue: lung tumor nodules were more in number in the LV-control group than those in the LV-MBNL2 group (Fig. 5e). The levels of MBNL2 in the lung tissue also increased in MBNL2-transduced mice, suggesting that overexpression of MBNL2 may have been responsible for the inhibition of lung metastasis in the mouse tumor model (Fig. 5e lower panel). These in vivo and in vitro results show that MBNL2 plays an important role in BC and LC metastasis.

### MBNL2 overexpression enhances the metastasis inhibition effect of NBT

**Fig. 6:**
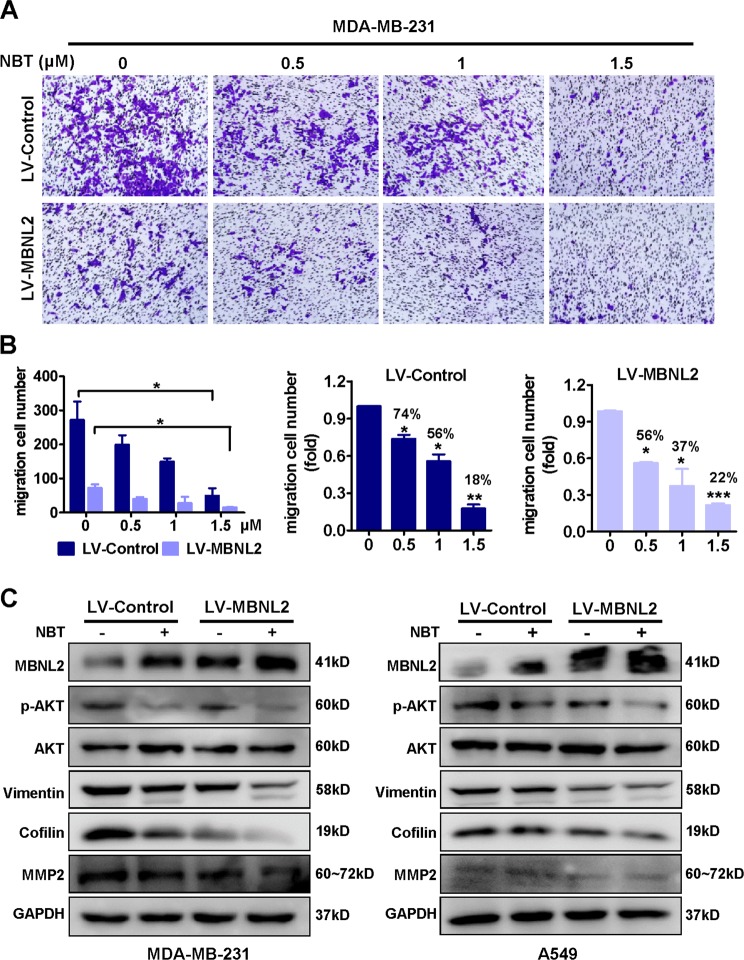
Overexpression of MBNL2 plus NBT inhibits metastasis in MDA-MB-231 cells. **a** Detection of cell migration using a transwell assay. MDA-MB-231 cells were transduced with lentiviruses expressing either MBNL2 plasmid or empty vehicle, and the cells were treated with various concentrations of NBT (0, 0.5, 1, 1.5 μM) for 24 h. After the assay, the cells were stained with crystal violet. **b** Results from the transwell assay. Cell numbers were counted in each group from three independent experiments. **c** Western blots of MDA-MB-231 and A549 cells transduced with lentiviruses expressing MBNL2 plasmid. The cells were treated with or without 2.5 μM NBT for 8 h, and the samples were analyzed for cofilin, pAKT, vimentin, MMP-2, MMP-9, and GAPDH. All experiments were performed in triplicate, and data are expressed as the mean ± SD of at least three independent experiments. *P < 0.05, **P < 0.01, ***P < 0.001

**Fig. 7:**
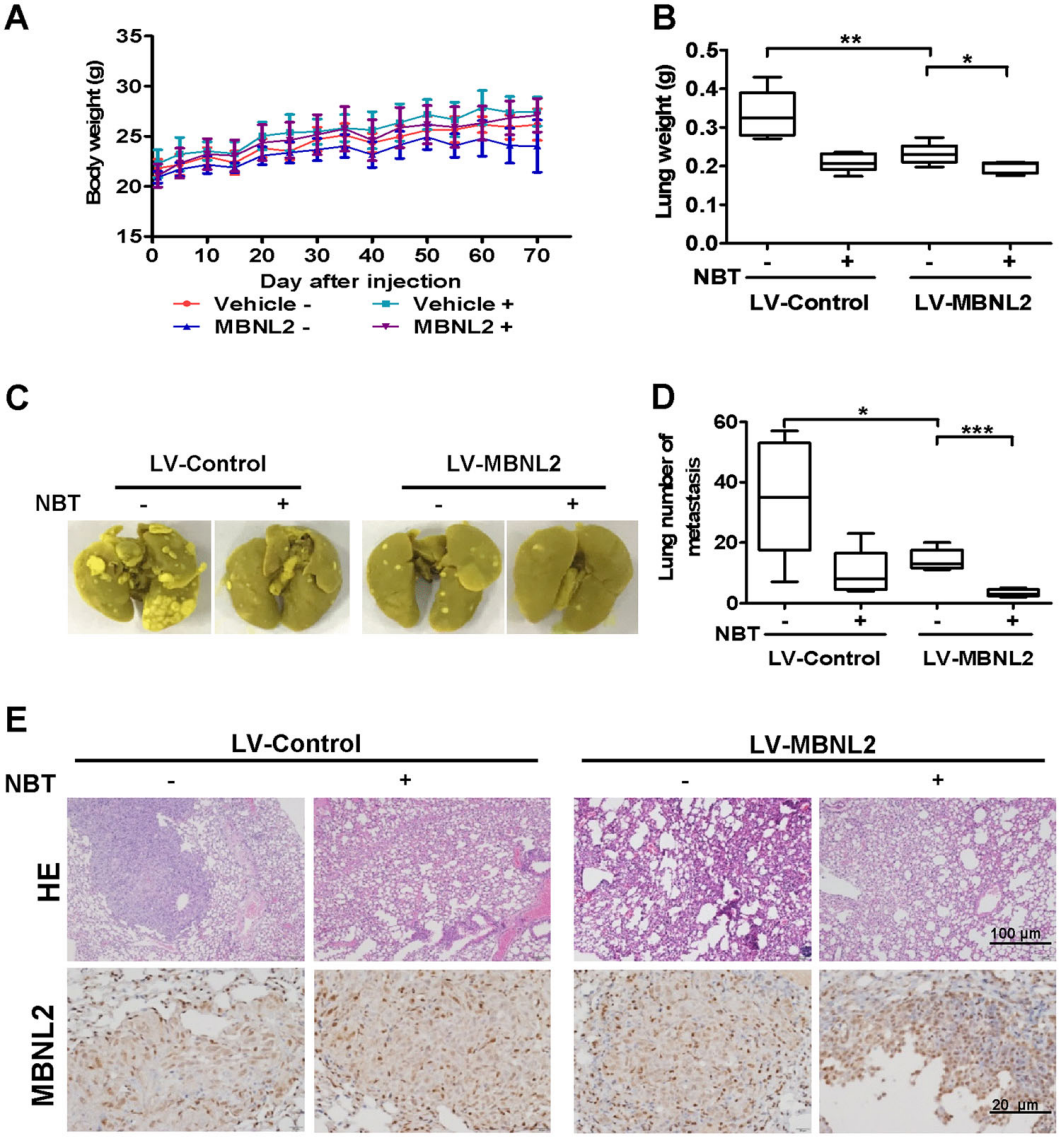
Overexpression of MBNL2 plus NBT suppresses metastasis in vivo. **a** The body weight of each group (*n* = 6) animals after injection of cells. After 2 months, the mice were killed, and lung weights were measured (**b**). Representative lung images taken from mice (**c**), and the number of tumor nodules (**d**). **e** Representative images of HE and IHC staining for MBNL2 of resected tumors. *P < 0.05, **P < 0.01, ***P < 0.001

Since NBT significantly inhibited tumor metastasis and increased the expression of MBNL2, we assumed that MBNL2 was partially responsible for the inhibitory effect of NBT on tumor metastasis. To verify this hypothesis, we first took advantage of MBNL2-overexpressing MDA-MB-231 cell lines (Fig. 4a, b). As shown in Fig. 6a, b, MBNL2 overexpression significantly enhanced the inhibitory effect of NBT treatment on tumor cell migration in MDA-MB-231 cells compared to controls transduced with the empty vector. To understand the potential mechanism, we assessed the expression of MBNL2 and other metastasis-related proteins. Upon NBT treatment

the expression of MBNL2 was significantly increased and the expression of pAKT, Vimentin, cofilin, and MMP2 decreased more in MDA-MB-231 cells overexpressing MBNL2 than in cells transfected with empty vector (Fig. 6c and Fig. S3d). These results suggested that MBNL2 could enhance the effect of NBT on the metastasis inhibition in MDA-MB-231 cells in vitro. As shown in Fig. S4a–c, the silencing of MBNL2 could partially eliminates the inhibitory effect of NBT on metastasis. We also established a mouse pulmonary metastasis model using MDA-MB-231 cells infected with either LV-control or LV-MBNL2. As shown in Fig. 7a, there were no significant differences in body weights between the vehicle and NBT treatment groups, but the metastatic nodules on the surface of the mouse lungs were dramatically decreased after overexpression of MBNL2 in the NBT-treated group compared to the control group, as demonstrated by the substantially reduced lung tumor nodules and weight (Fig. 7b−d). The tissues in mouse, such as heart, liver, spleen, and kidney, which did not show obvious morphological changes (Fig. S5b), suggesting The side effects of NBT would be small. The lungs were resected and examined by HE and IHC staining. Tumor tissues derived from LV-MBNL2 transduced cells after NBT treatment exhibited an increase in the MBNL2 protein compared with the control groups (Fig. 7e). Therefore, MBNL2 overexpression enhanced the inhibitory effects of NBT on BC metastasis in vitro and in vivo. In summary, MBNL2 overexpression combined with NBT inhibited metastasis in the breast tumor in vitro and in vivo to a larger extent than either treatment alone.

## Discussion

Cancer metastasis is responsible for the vast majority of cancer-related mortality and remains a major hurdle in cancer therapy, which warrants the development of more specific approaches targeting metastasis. In this work, we examined a number of indicators for metastatic function and found that NBT, a natural compound isolated from *Garcinia bracteata*, has a potent inhibitory effect on the metastasis of BC and LC.

Post-transcriptional regulation, known to be an essential regulator of gene expression, participates in numerous physiological and pathological processes and is an important feature of metastatic cancer^18,22^. According to our previous RNA-seq analysis, the expression of more than 2000 genes was affected differentially by NBT, and further bioinformatics analysis showed that NBT could affect RNA-binding proteins (RBPs) to regulate alternative splicing (AS) events (data not shown). As one of the post-transcriptional gene regulatory mechanisms, AS enables a single gene to produce multiple messenger RNA variants and distinct protein isoforms, sometimes with different or even opposing roles in the development of tumors, such as proliferation, apoptosis, angiogenesis, drug resistance, and metastasis^23–25^. RNA-binding proteins, which are involved in the AS process, have many functions in biologic processes, deregulation of RBP affects every step of cancer development, such as sustained cell proliferation, evasion of apoptosis, avoiding immune surveillance, inducing angiogenesis, and activating metastasis^26,27^; mounting evidence supports a vital role for RBPs in tumor growth and metastasis^28–30^.

In this study, we showed that the RNA-binding protein MBNL2 was upregulated after treatment with NBT. MBNL2 was first identified as a regulator of muscular dystrophy^31–33^, while its function in cancer was rarely examined until a recent study. Based on deep sequencing analysis of the transcriptomes of epithelial and mesenchymal cells, results from previous study revealed that MBNL2 altered the splicing of EMT-associated regulators and suppressed cell proliferation, tumorsphere formation, and migration in hepatocellular carcinoma in vivo^17,34^. Based on the inhibitory effect of NBT on tumor metastasis and MBNL2 upregulation, we hypothesized that the inhibition of tumor metastasis by NBT was associated with the upregulation of the MBNL2 expression. Analysis of TCGA database showed that the expression of MBNL2 in tumor tissues from BC and LC patients was significantly downregulated compared with that in normal tissues, indicating an inhibitory role for MBNL2 in tumor metastasis. Further in vivo and in vitro studies confirmed that MBNL2 overexpression significantly inhibited the migration of BC and LC cells, an effect similar to NBT intervention. Furthermore, inhibition of cancer cell migration by NBT was enhanced when MBNL2 was overexpressed, and silencing of MBNL2 could partially eliminates the inhibitory effect of NBT on metastasis. In summary, we have provided evidence that MBNL2 may act as a potential regulator of tumor metastasis and a mediator of the effects of NBT.

Mechanistically, we demonstrated that overexpression of MBNL2 downregulated phospho-AKT and other key proteins involved in cell metastasis, including the EMT marker vimentin and cofilin. AKT was reported to be a nodal oncoprotein critical for regulating cell survival and metastasis of cancer cells^35,36^, and vimentin, as one of the important biomarkers of EMT, both crucial in the initiation and promotion of tumor metastasis^37^. Cofilin is a key regulator of actin dynamics in the tumor microenvironment that dominates cell adhesion^38^. Therefore, upregulation of MBNL2 by NBT may exert anti-cancer metastasis activity by affecting multiple targets such as phospho-AKT and EMT transformation. However, the specifics of the MBNL2 action on those potential targets will need to be investigated in future studies.

Tumor metastasis is a multistep process involving metastasis formation and colonization. Approximately one-third of BC patients harbor metastases in lymph nodes at the time of surgery, which highlights the necessity for drugs targeting metastatic colonization^39^. Our work found that NBT impeded BC and LC cell migration and extravasation into the lung parenchyma, thereby revealing a potential capacity to inhibit the formation of metastasis. Although further studies are needed to verify these data, our results, to a certain extent, suggest that NBT may inhibit metastatic seeding and outgrowth.

In summary, our study identified a compound (NBT), which prevents metastasis. Our results demonstrated that NBT blocks BC and LC metastasis through targeting MBNL2 and provided insights into the mechanism of action for the inhibitory effect of MBNL2 on metastasis. We showed the role of MBNL2 in tumor metastasis, which raises the possibility of assessing the clinical association between MBNL2 and metastasis. These results provide opportunities for developing MBNL2-targeted agents using NBT as a lead compound.

## Materials and methods

### Cell cultures

The BC cell lines MDA-MB-231, MCF-7, and LC cell lines A549, H1650, H2228 were obtained from the Shanghai Institute of Biochemistry and Cell Biology (Shanghai, China), and human umbilical vein endothelial cells (HUVEC) was kindly provided by Dr. Xiaohua Yang, Shanghai Chest Hospital. Cells were maintained in DMEM (Sigma, D11527) supplemented with 10% fetal bovine serum (FBS) (Biological Industries, 04-001-1ACS, Beit HaEmek, Israel) and 100 U/mL penicillin-streptomycin (Gibco/Invitrogen, 15140-122, Carlsbad, CA, USA). Cells were cultured in a standard humidified atmosphere of 5% CO_2_ at 37 °C.

### **C**ell viability assay

The cells were seeding in 96-well plate and exposed to NBT for 24–48 h. Cell viability was measured using the MTT reagent (3-(4,5-dimethylthiazol-2-thiazolyl)-2,5-diphenyltetrazolium bromide) in Cell Proliferation Reagent Kit I (Sigma), 10 μL MTT solution was added to each well of a 96-well plate and incubated for 4 h at 37 °C. After the medium was removed, 100 μL aliquots of DMSO were added to dissolve the purple crystals. After shaking for 5 min, the absorbance was measured at 490 nm using a microplate reader (FLUOstar Omega, BMG Labtech, Germany). The IC_50_ values were calculated from dose–response curves using GraphPad Prism 5 (La Jolla, California, USA).

### Wound healing and cell migration assays

Wound healing and cell migration assays were used to evaluate cell motility. In the wound healing assay, cancer cells were seeded into a 24-well culture plate. When the cells grew to 90% confluence, a scratch was gently created through the cell monolayer by sterile 100 μL pipette tips, and loose cells were washed away. The cell migration was observed and imaged under an IX83 microscope for each condition and timepoint (Olympus, Tokyo, Japan). cell migration was estimated using transwell chambers (Millicell, Germany) with a pore size of 8 μm. Then, 3 × 10^4^ cells resuspended in 100 μL serum-free medium were seeded in the upper chamber, with serum-containing medium in the lower chamber of 24-well transwell plates (BD Biosciences, San Jose, CA). After 24 h, the experiment was terminated by wiping the cells from the wells with a cotton swab, fixing and staining with 0.05% crystal violet for 20 min, and scoring under a light microscope in five random fields.

### Western blotting analysis

Cells were harvested and lysed in RIPA buffer, and proteins were separated on SDS polyacrylamide gels and transferred to PVDF membranes (Millipore, Billerica, MA, USA). The membranes were blocked with 5% nonfat milk, then immunoblotted with primary antibodies (Supplementary Table 1) at 4 °C overnight. After being washed three times with TBST, membranes were incubated for 1 h with appropriate secondary antibodies at room temperature. GAPDH was used as the loading control. Membranes were visualized with ImageQuant LAS 4000 (General Electric Company).

### RNA extraction and quantitative real-time PCR

RNAs were extracted using Trizol Reagent (Beyotime, R0016), and reverse transcription was carried out using 2 μg RNA in a 20 μL reaction volume using the PrimeScript RT reagent kit (Takara Biotechnology, China). Quantitative PCR was conducted with SYBR Green for detection (SYBR® Premix Ex TaqTM) in 20 μL reactions using the ABI PRISM® 7500 Real-Time PCR System (Applied Biosystems, Grand Island, NY, USA). GAPDH was used as an internal control. The primers for human genes are presented in Supplementary Table 2.

### Immunofluorescence assay

Cells were plated on coverslips, and incubated at 4 °C overnight and exposure to 2.5 μM NBT for 24 h. Then, the cells were fixed with 4% paraformaldehyde/PBS, blocked with 10% BSA in PBS and incubated with antibodies specific for MBNL2 (1:100), followed by Cy3-labeled secondary antibody(Beyotime, Shanghai, China). and then stained with 4′,6-diamidino-2-phenylindole (DAPI). Labeled cells were visualized on an inverted fluorescent microscope (Olympus, Japan).

### Plasmid transfection and lentiviral infection

The DNA fragment representing the open reading frame of MBNL2 (Supplementary Table 3) was purchased from Convenience Biology (Changzhou, China), subcloned into the lentiviral vector pPLVX-IRES-ZsGreen1 (Convenience Biology, Changzhou, China), and the expression vector was subsequently cotransfected with a 2nd Generation Packaging System Mix (Lenti-Pac^TM^ HIV Expression Packaging Kit, GeneCopoeia, Changzhou, China) into the HEK 293T lentiviral package cells to produce lentiviral particles.

### Construction of stable cell lines

To obtain cell lines stably overexpressing MBNL2, the target cells MDA-MB-231 and A549 were infected with the LV-MBNL2 and LV-control viruses; after incubating with viruses for 72 h, the cells were harvested, washed with PBS, and resuspended with 20 μg/mL DNase (1453GR001 BioFroxx, Germany) and 1% gentamicin (1121MG010 BioFroxx, Germany). A flow cytometer (Beckman Moflo, Germany) was used to screen for cells overexpressing MBNL2. The positive cells of overexpressing MBNL2 was harvested and incubated and the infection efficiency was confirmed by western blotting and qRT-PCR.

### RNA interference

MBNL2 siRNA and negative control siRNA (Convenience Biology, Changzhou, China) were transfected with Lipofectamine RNAiMAX Reagents (Invitrogen) according to the manufacturer’s instructions. The oligonucleotide sequences were listed in Supplementary Table 2.

### Colony formation assays

Cells were cultured in six-well plate in a medium for 14 days, the colonies were fixed with methanol for 15 min and stained with 1% crystal violet for 20 min. The observable colonies were manually tallied.

### Tail vein injections in nude mice

We purchased female BALB/C nude mice (4–5-weeks-old) from the Experimental Animal Center of the Chinese Academy of Science, Shanghai, China, and used them for the study of pulmonary metastasis following the injection of cancer cells into the tail vein (PZSHUTCM18111603). We injected cells of the breast cancer line MDA-MB-231 (1 × 10^6^ cells) intravenously into 6-week-old female nude mice. The animals were then divided into three groups (*n* = 8 in each group), and DMSO, NBT (2.5 mg/kg), or 5-FU (20 mg/kg) were administered by an intraperitoneal injection every other day. In another test, MDA-MB-231 cells stably transfected either with MBNL2 or the empty vector were resuspended at 3 × 10^7^ cells/mL. A suspension of 6 × 10^6^ cells in a volume of 200 μL was injected intravenously into female nude mice. Body weight was measured every second day. Two months later, the mice were sacrificed, and the lungs removed and fixed in Bouin’s solution and 4% paraformaldehyde for hematoxylin and eosin (HE) staining and immunohistochemistry (IHC).

### Immunohistochemistry

Tumors were fixed in 10% neutral-buffered paraformaldehyde. Next, the samples were embedded in paraffin, stained with hematoxylin and eosin, MBNL2 (Santa Cruz, Cat. sc-136167). Finally, the sections were mounted with DPX Mountant (Sigma, 317616) for histological analysis.

### Statistical analysis

Data are presented as the mean ± S.D. Student’s *t-*test was used for comparison between the two different groups, and one-way analysis of variance (ANOVA) was used for the multiple comparisons. A two-tailed value of *P* < 0.05 was considered statistically significant. Statistical analyses were carried out using GraphPad Prism 5.0.

## Supplementary information

supplementary figure legends.

Supplementary Tables.

supplementary figure 1.

supplementary figure 2.

supplementary figure 3.

supplementary figure 4.

supplementary figure 5.
