# Supplementary material for: The natural compound neobractatin inhibits tumor metastasis by upregulating the RNA-binding-protein MBNL2
Source: Cell Death Dis. 2019 Jul 18;10(8):554. doi: 10.1038/s41419-019-1789-5 (PMC6639345; doi:10.1038/s41419-019-1789-5)
Supplement: Supplementary file 1 — supplementary figure legends. [file 41419_2019_1789_MOESM1_ESM.docx]

**Supplementary figure legends**

**Figure S1. NBT inhibits AKT phosphorylation and regulates key proteins involved in cell metastasis.** **a.** The statistic analysis of relative protein level from Figure 1f. Data shown are mean ± SD of three independent experiments. *P < 0.05, **P < 0.01, ***P < 0.001. **b**. AKT phosphorylation and epithelial-mesenchymal transition (EMT) proteins were analyzed by western blot. MDA-MB-231 and A549 cells treated with various concentrations of NBT (0, 0.5 μM, 1 μM, 1.5 μM, 2 μM, 2.5 μM) for 8 h. The statistic analysis from three independent experiments was shown in the lower panel. *P < 0.05, **P < 0.01

**Figure S2. NBT increases the expression of MBNL2**. **a.** MDA-MB-231 and A549 cells were treated with various concentrations of NBT (0, 0.5 μM, 1 μM, 1.5 μM, 2 μM, 2.5 μM) for 8 h. The statistic analysis from three independent experiments was shown in the lower panel, *P < 0.05, **P < 0.01. **b.** Immunohistochemistry staining of MBNL2 in lung tissue after NBT and 5-FU treatment. **c.** Immunofluorescence analysis of A549 cells using MBNL2 antibody. The cells were treated with 2.5 μM NBT for 24 h and detected with His-tag MBNL2 antibody using a microscope. (Scale bar: 20 *μ*m). Enlarged images are shown in the right panel of each sample.

**Figure S3. Overexpression of MBNL2 suppresses cancer metastasis.** **a.** A colony formation assay of MDA-MB-231 and A549 cells transduced with lentiviruses expressing the indicated MBNL2. Cells were grown for 14 days, then stained with crystal violet. **b.** Colony counts and images of the cells. The data represent the mean ± S.D. from three independent experiments. NS non-significant **c-d.** The statistic analysis of relative protein level from Figure 4e and Figure 6c . Data shown are mean ± SD of three independent experiments. *P < 0.05, **P < 0.01, ***P < 0.001.

**Figure S4**. **MBNL2 knockdown partially eliminates the inhibitory effect of NBT on metastasis**. **a.** MDA-MB-231 and A549 cells were transfected with scramble siRNA or individual siRNAs (No. 1 to 2) to MBNL2 for 48h analyzed by western blotting. **b.** Transwell assay. MDA-MB-231 cells were transfected with MBNL2 siRNA (#2), and 48 h after transfection, the cells were treated with or without 1.5 μM NBT for 24 h, detected using a transwell assay and stained with crystal violet. **c.** The cell number from transwell assay was counted in each group from three independent experiments. *P < 0.05, **P < 0.01.

**Figure S5.** **NBT exhibits low toxicity *in vivo***. Six-week-old nude mice were treated with NBT for 2 months. **a**. Images of heart, liver, spleen, and kidney from nude mice treated with the vehicle, NBT (2.5 mg/kg), or 5-FU (20 mg/kg). **b**. Images of heart, liver, spleen, and kidney from vehicle- and NBT (2.5 mg/kg)-treated nude mice, with and without transduction with LV-control and LV-MBNL2.
