# Supplementary material for: The natural compound neobractatin inhibits tumor metastasis by upregulating the RNA-binding-protein MBNL2
Source: Cell Death Dis. 2019 Jul 18;10(8):554. doi: 10.1038/s41419-019-1789-5 (PMC6639345; doi:10.1038/s41419-019-1789-5)
Supplement: Supplementary file 2 — Supplementary Tables. [file 41419_2019_1789_MOESM2_ESM.docx]

**Supplementary Tables**

**Supplementary Table 1. Primary antibodies used in this study**

| **Antibody (Item No.)** | **Specificity** | | |  | **Company** | | |
| --- | --- | --- | --- | --- | --- | --- | --- |
|  | **WB** | **IHC** | **IF** | |  |  |  |
| p-AKT (4060) | 1:1000 |  |  | | Rabbit monoclonal | Cell Signaling Technology |  |
| AKT (9272) | 1:1000 |  |  | | Rabbit polyclonal | Cell Signaling Technology |  |
| Vimentin (5741) | 1:1000 |  |  | | Rabbit monoclonal | Cell Signaling Technology |  |
| Cofilin (5175) | 1:1000 |  |  | | Rabbit monoclonal | Cell Signaling Technology |  |
| MMP-2 (10373-2-AP) | 1:1000 |  |  | | Rabbit monoclonal | Proteintech |  |
| GAPDH (5174) | 1:1000 |  |  | | Rabbit monoclonal | Abcam |  |
| MBNL2 (sc-136167) | 1:200 | 1:100 | 1:100 | | Mouse monoclonal | Santa Cruz |  |
| MBNL1 (sc-47740) | 1:200 |  |  | | Mouse monoclonal | Santa Cruz |  |
| MBNL3 (EPR16337, ab197590) | 1:1000 |  |  | | Rabbit monoclonal | Abcam |  |
| β-actin (S473) | 1:1000 |  |  | | Rabbit monoclonal | Cell Signaling Technology |  |
|  |  |  |  | |  |  |  |

**Supplementary Table 2. Primer sequences and target sequences used in this study**

| **Gene** | **Sequence or Target Sequence** |
| --- | --- |
| MBNL2-F | 5’-CAGAAACGGAATGGAATG-3’ |
| MBNL2-R | 5’-TTGAGGAGGAAACAGGATAG-3’ |
| MBNL2 isoform 1-F | 5’- TACCTCCAGTTTGTCAGTCC-3’ |
| MBNL2 isoform 1-R | 5’- ATTAGCTTTGCTCCTTACCT-3’ |
| MBNL2 isoform 3-F | 5’- ACTCCCTGCATACCTCCA-3’ |
| MBNL2 isoform 3-R | 5’- CACCACGCCTGTTATTGT-3’ |
| MBNL2 isoform 4-F | 5’- AAAGCGGCAGTCGGTCTC-3’ |
| MBNL2 isoform 4-R | 5’- CCCTGGAGTTGGGTTGGT-3’ |
| Vimentin-F | 5’-CGTGATGCTGAGAAGTTTCGT-3’ |
| Vimentin-R | 5’-TGGATTCACTCCCTCTGGTTG-3’ |
| Snail-F | 5’-GCCTTTCCCACTGTCCTCATC-3’ |
| Snail-R | 5’-AGTTTACCTTCCAGCAGCCCTAC-3’ |
| Cofilin-F | 5’- TGCTGCCAGATAAGGACTGC-3’ |
| Cofilin-R | 5’- CTCTTAAGGGGCGCAGACTC-3’ |
| MMP2-F | 5’-GTGCTGAAGGACACACTAA-3’ |
| MMP2-R | 5’-TTGCGAGGGAAGAAGTTG-3’ |
| MMP9-F | 5’-TTTGACAGCGACAAGAAGT-3’ |
| MMP9-R | 5’-CTCAGTGAAGCGGTACATA-3’ |
| GAPDH-F | 5’-ACGACCACTTTGTCAAGCTC-3’ |
| GAPDH-R | 5’-GTTGCTGTAGCCAAATTCGT-3’ |
| MBNL2 siRNA -1 | 5'-GCTCTGATGAAGAATGCAAATTTGCTCAT-3' |
| MBNL2 siRNA -2 | 5'-CGGCTATTAGCTTTGCTCCTT-3' |
| Control siRNA | 5'-GGGTGAACTCACGTCAGAA-3' |
|  |  |
|  |  |

**Supplementary Table 3. Plasmid Map-sequence of MBNL2**

Gene Name： MBNL2

| Gene Sequence |
| --- |
| GAATTCGCCACCATGGCTTTGAACGTTGCCCCAGTCAGAGATACAAAATGGCTGACATTAGAAGTCTGCAGACAGTTTCAAAGAGGAACATGCTCACGCTCTGATGAAGAATGCAAATTTGCTCATCCCCCCAAAAGTTGTCAGGTTGAAAATGGAAGAGTAATTGCCTGCTTTGATTCCCTAAAGGGCCGTTGTTCGAGAGAGAACTGCAAGTATCTTCACCCTCCGACACACTTAAAAACTCAACTAGAAATTAATGGAAGGAACAATTTGATTCAGCAAAAAACTGCAGCAGCAATGCTTGCCCAGCAGATGCAATTTATGTTTCCAGGAACACCACTTCATCCAGTGCCCACTTTCCCTGTAGGTCCCGCGATAGGGACAAATACGGCTATTAGCTTTGCTCCTTACCTAGCACCTGTAACCCCTGGAGTTGGGTTGGTCCCAACGGAAATTCTGCCCACCACGCCTGTTATTGTTCCCGGAAGTCCACCGGTCACTGTCCCGGGCTCAACTGCAACTCAGAAACTTCTCAGGACTGACAAACTGGAGGTATGCAGGGAGTTCCAGCGAGGAAACTGTGCCCGGGGAGAGACCGACTGCCGCTTTGCACACCCCGCAGACAGCACCATGATCGACACAAGTGACAACACCGTAACCGTTTGTATGGATTACATAAAGGGGCGTTGCATGAGGGAGAAATGCAAATATTTTCACCCTCCTGCACACTTGCAGGCCAAAATCAAAGCTGCGCAGCACCAAGCCAACCAAGCTGCGGTGGCCGCCCAGGCAGCCGCGGCCGCGGCCACAGTCATGGCCTTTCCCCCTGGTGCTCTTCATCCTTTACCAAAGAGACAAGCACTTGAAAAAAGCAATGGTACCAGCGCGGTCTTTAACCCCAGCGTCTTGCACTACCAGCAGGCTCTCACCAGCGCACAGTTGCAGCAACACGCCGCGTTCATTCCAACAGGGTCAGTTTTGTGCATGACACCCGCTACCAGTATTGATAATTCTGAAATAATCAGCAGAAACGGAATGGAATGCCAAGAATCTGCATTGAGAATAACTAAACATTGTTACTGTACATACTATCCTGTTTCCTCCTCAATAGAATTGCCACAAACTGCATGCGATTACAAGGATGACGACGATAAGTAAGGATCC |
|  |

**Vector Name：PLVX-IRES-ZsGreen1.**

**Clone site：EcoRI – BamHI.**
